# Respiratory and limb muscles’ ability to repeatedly generate maximal isometric strength in patients with intensive care unit-acquired weakness: an observational study

**DOI:** 10.1186/s12871-025-03008-y

**Published:** 2025-03-20

**Authors:** Margaux Machefert, Guillaume Prieur, Solène Aubry, Yann Combret, Clément Medrinal

**Affiliations:** 1 Physiotherapy Department, Le Havre Hospital, Le Havre, F-76600 France; 2 Paris-Saclay University, UVSQ, ERPHAN UR 20201, Versailles, F-78000 France; 3 Intensive Care Department, Le Havre Hospital, Le Havre, F-76600 France; 4 Private practice, 92 Boulevard des États-Unis, Le Vésinet, F-78110 France; 5 Institute for Research and Innovation in Biomedicine (IRIB), Univ Rouen Normandie, GRHVN UR 3830, Rouen, F-76000 France

**Keywords:** Intensive care unit-acquired weakness, Muscle weakness, Quadriceps muscle, Inspiratory muscles

## Abstract

**Background:**

Intensive care unit-acquired weakness (ICU-Aw) is a prevalent complication in critically ill patients, affecting both limb and respiratory muscles, individually or concurrently. The precise mechanisms by which muscle weakness influences the distinct functional roles of each muscle group remain to be fully elucidated. The objective of this study was to compare the time course evolution of inspiratory and quadriceps muscles strength during repeated maximal isometric contractions in patients with limb muscles and inspiratory muscles weakness.

**Methods:**

A single-center, observational study was conducted in critically ill patients after extubation, presenting with both inspiratory and limb muscle weakness (defined as maximal inspiratory pressure (Pi_max_) < 30 cmH_2_O and an MRC score < 48). The patients’ ability to sustain maximal voluntary effort was measured using electronic manometers and dynamometers, with repeated efforts performed 10 times. Following each measurement, a 10-second rest period was observed, and strength measurements were repeated to evaluate recovery.

**Results:**

A total of 20 patients (90% male, mean age 61 ± 10 years, SAPS II score 28 ± 17) were included. The mean first maximal inspiratory pressure was 32.6 ± 17 cmH_2_O, and the mean first quadriceps maximal force was 135 ± 90 Newtons (N). Investigation revealed a decline in quadriceps muscle force of -15.45 ± 28.61 N (95% CI: -28.84 to -2.05) while inspiratory muscles demonstrated stability (mean difference: 1.75 ± 7.57 cmH_2_O (95% CI: -1.80 to 5.30)). A statistically significant interaction between time and muscle group was identified (*p* = 0.0017), suggesting a different time course evolution of maximal voluntary strength between muscle groups. After a one-minute recovery, significant improvement in quadriceps strength was observed (*p* = 0.009), while no statistically significant change was detected in inspiratory muscle strength (*p* = 0.16).

**Conclusions:**

The results of this study indicate potential disparities in the maximum force maintenance capacity between the quadriceps muscles and inspiratory muscles in patients with ICU-acquired weakness.

**Trial registration:**

Registered on ClinicalTrials.gov Identifier NCT05396066.

**Supplementary Information:**

The online version contains supplementary material available at 10.1186/s12871-025-03008-y.

## Introduction

Intensive care unit-acquired weakness (ICU-AW) is a complex multifactorial condition affecting both respiratory and limb muscles, sometimes individually or concurrently [1, 2]. This condition is linked to inflammation, oxidative stress, metabolic and neural dysfunctions [3], and affects between 25 and 75% of ICU survivors depending on definitions, assessment time, diagnostic methods, and study populations, with a mean of 40% [4, 5]. Key risk factors include mechanical ventilation, sedation, prolonged inflammatory status, multi-organ failure, bed rest, and ICU length of stay [1, 6–9]. ICU-AW carries a poor prognosis and is associated with difficulties in weaning from mechanical ventilation, prolonged hospitalization, increased ICU mortality, a decline in functional independence at discharge and up to 5 years after hospital release [6, 10–12].

Despite its prevalence, the underlying mechanisms of ICU-AW remain unclear. As respiratory muscle weakness appears to be twice as common as limb muscle weakness in intensive care patients [13], and doesn’t have the same prognosis, is not clear whether ICU-AW represents a single syndrome or two distinct syndromes affecting two different types of muscles, both respiratory and limb muscles [10, 14, 15]. While these muscle groups do share common features and cellular pathways [16, 17], they also function differently and may not be similarly affected by mechanical ventilation and bed rest [8, 14]. Given their significant impact on ICU survivors’ outcomes, understanding the differences between respiratory and limb muscle involvement and recovery could guide our rehabilitation programs in ICU [18, 19].

Inspiratory muscles are designed to sustain repeated contractions, and their oxidative physiological characteristics [20], along with their continuous action in healthy individuals, could allow them to maintain greater capacity, unlike limb muscles. While the diaphragm is the primary inspiratory muscle, other muscles such as the external intercostals, scalenes and sternocleidomastoids also contribute to inspiratory effort.

This aim of this study was to investigate and compare the ability of the inspiratory and quadriceps muscles to repeatedly produce maximal isometric voluntary force in patients with intensive care unit- acquired weakness with both types of muscle weakness at extubation. The study hypothesis was that the inspiratory muscles would show greater resistance to repeated maximal contractions compared with the quadriceps muscle.

## Materials and methods

### Study design

We conducted a single-center observational trial in Le Havre Hospital from January to December 2023. Ethical approval was granted by the French Comité de Protection des Personnes Nord Ouest IV (N°21.03849.000040), and informed consent was obtained from all participants or their relatives. The study was prospectively registered on www.clinicaltrials.gov (NCT05396066). The manuscript as a supplemental data document.

### Eligibility and enrolment

Patients fulfilling the following criteria at eligibility assessment were included: (1) critically ill patients aged 18 or more, (2) weaned from mechanical ventilation (i.e. extubated or tracheostomised and decannulated), (3) presenting limb and inspiratory muscle weakness (i.e. MRC score < 48 and Pi_max_ <30 H_2_0 at the time of extubation), (4) able to stand with help (ICU Mobility Scale 4). This last point was chosen for practical and safety reasons: we assumed patients would need time from mechanical ventilation weaning to be able to understand and perform maximal isometric volitional contractions. Non-inclusion criteria were: patients with neurological pathology history or pre-existing neuromuscular dysfunction, patients unable to walk 50 m before ICU admission, patients with thoracic or abdominal surgery, and guardianship. Patients were excluded if they were unable to carry out voluntary contractions.

### Interventions

A daily assessment for inclusion was made in the unit. Participants were included when weaned from mechanical ventilation and if presenting both types of weaknesses. When they could stand up with help, strength measurements were undertaken by a single physiotherapist following the European or American Thoracic Society’s recommendations [21, 22]. We used a manual hand-held dynamometer (Microfet 2^®^, Biometrics) to measure the quadriceps muscle’s strength and an external electric manometer with a unidirectional valve (MicroRPM^®^, Carefusion) to measure the inspiratory muscles’ strength. Both quadriceps and inspiratory muscle measurements were made on the same day, always in this specific order.

#### Quadriceps muscle strength

Quadriceps maximal isometric volitional strength was measured on a sitting patient with knees and hips in 90° of flexion, foot not touching the ground, as recommended [22]. A strap was positioned at the lower end of the leg segment, above lateral malleolus, on the anterior side, and directly connected to the dynamometer attached to the chair. Before the test, patients were trained in the protocol with 3 to 5 repetitions, to familiarize them with the instructions. Patients were asked to provide a maximal effort against the strap and were vigorously encouraged to maintain the contraction for 3 to 5 s. The measurements were repeated until a difference of less than 10% was obtained for a series of measurements. After a 5-minute break to let patients recover, participants performed the set of 10 repetitions of the same maximal isometric effort, separated by a 10-secondes break. One minute after stopping the effort, maximal muscle strength was measured again. Quadriceps muscle force was reported in Newton (N) and expressed as a percentage of the first measurement of the set.

#### Inspiratory muscles strength

Maximal inspiratory pressure (Pi_max_) was measured in the same sitting upright position, lips clamped around the dynamometer’s mouthpiece, and nose clip in place, at residual volume, as recommended [21]. Participants were given the same explanation and encouragement as for the quadriceps measurements, except they needed to fully breathe out before inhaling as hard as they could. Measurements were carried out in a similar way, using the same protocol. Pimax was measured in centimetres of water (cmH_2_O) and expressed as a percentage of the first measurement of the set.

### Characteristics and outcomes

At admission we collected demographic data, comorbidities, primary cause of admission, and illness severity by Simplified Acute Physiology Score (SAPS II). We collected maximal inspiratory pressure and Medical Research Council score the day patients were weaned off mechanical ventilation, enabling the presence of weaknesses to be established, and counted days from extubation to muscle force measurements.

### Statistical methods

Continuous data were expressed as means (± standard deviation, or SD) or medians (interquartile range) depending on the distribution, and categorical data were expressed as counts (%). Changes in outcome measures over the ten repetitions for both muscle groups were compared using a two-way analysis of variance (ANOVA) for repeated measures. Post-hoc comparisons were made between each time point and each group, and an exploratory analysis was performed to assess the impact of the delay between measurements and extubation on changes in muscle strength over time. Comparison of muscle force after one-minute recovery were assessed using paired Student’s t-tests. A *p*-value of less than 0.05 was considered statistically significant for all comparisons. Prism 8© software (GraphPad, California) was used for statistical analysis.

## Results

Twenty patients were included, mainly men (90%), with a mean age of 61 ± 10 years old and a SAPSII score of 28 ± 17. Patients included remained under mechanical ventilation for an average of 28 ± 14 days, and the mean ICU length of stay averaged 40 ± 18 days. Strength measurements were carried out on average 9 ± 4 days after weaning from mechanical ventilation (i.e. extubation) (see Table 1).

**Table 1:** Cohort characteristics

Variables	Total (*N* = 20)	
**At admission**		
Age, years	61 (10)	
Gender (male)	18 (90)	
Body Mass Index, kg/m²	27,4 (8,7)	
IGSII at ICU admission, ua	28 (17)	
**Main cause of admission**		
Acute respiratory failure	11 (55%)	
Post-surgery	5 (25%)	
Acute renal failure	2 (10%)	
Acute cardiac failure	1 (5%)	
Sepsis	1 (5%)	
**Co-morbidity**		
Chronic respiratory disease	3 (15%)	
Chronic cardiac insufficiency	4 (20%)	
Hypertension	4 (20%)	
Diabetes	4 (20%)	
Chronic kidney insufficiency	3 (15%)	
Hypercholesterolemia	4 (20%)	
Obesity (BMI ≥ 30)	5 (25%)	
**ICU stay**		
ICU length of stay (nb days)	40 (18)	
Nb days under MV	28 (14)	
MRC score at MV weaning	34 (7)	
Delay between weaning and measurements (nb days)	9 (4)	

Data are expressed at median (IQR) and at n (%)

OQR interquartile range; IGSII Index de Gravité Simplifié II; ICU Intensive Care Unit; ua unity arbitrary; BMI Body Mass Index; MV Mechanical Ventilation; MRC score Medical Research Council score

**Fig. 1:**
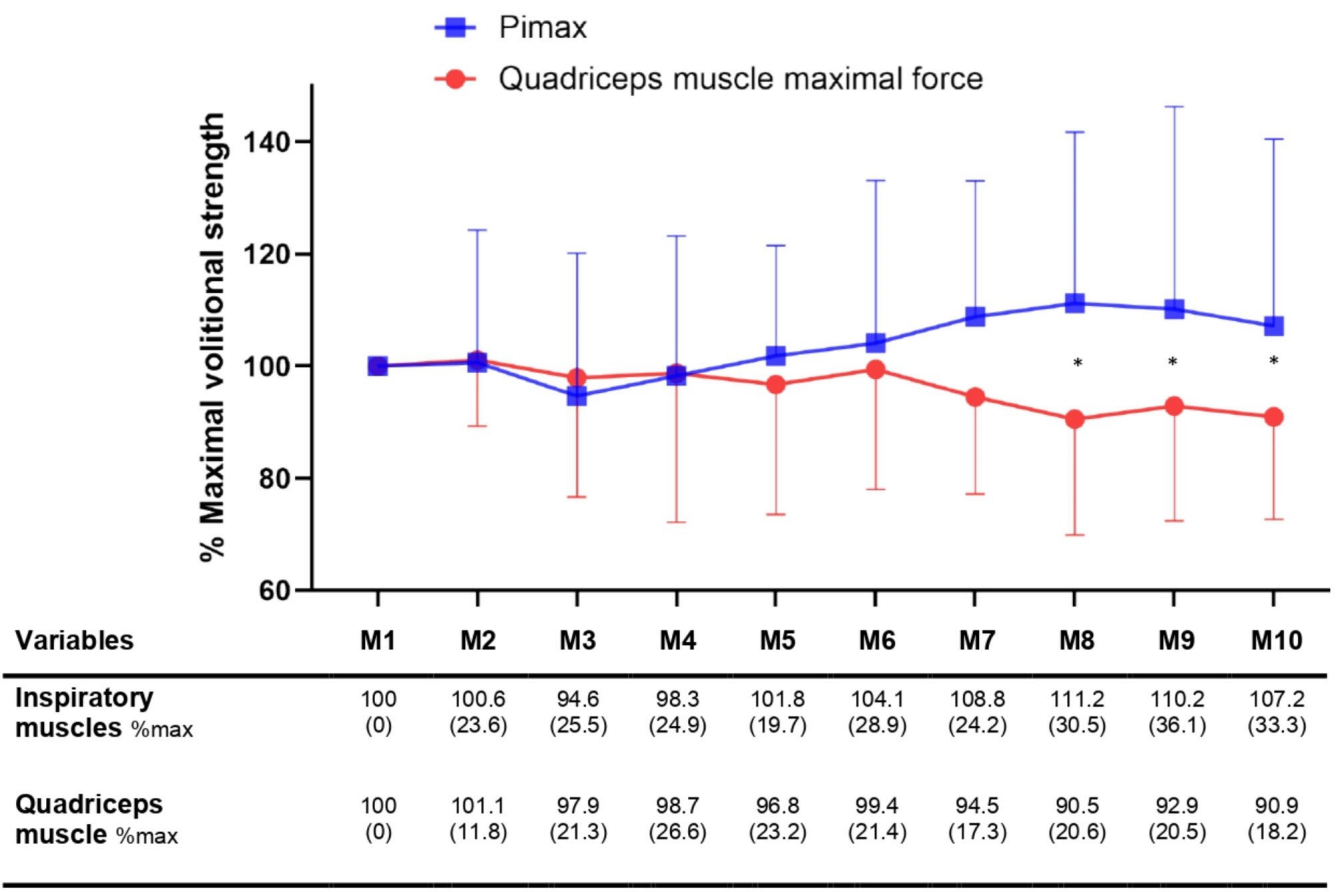
Muscle force time course evolution over the ten repetitions. Data are expressed as mean (± standard deviation). M1: measurement 1; Pimax: maximal inspiratory pressure. * indicates a post-hoc statistically significant between-group difference

On the day of the measurements, the mean Pimax was 32.6 ± 17 centimetres of water, and the quadriceps strength was 101.8 ± 64.4 Newtons. Over the ten repeated contractions, quadriceps muscle force decreased by -15.5 ± 28.6 N (95%CI: -28.8 to -2), equivalent to a loss of 9.07% ±18.24 (95%CI: -17.60 to -0.52) with no significant change for inspiratory muscles (mean difference: 1.75 ± 7.57 cmH_2_O (95%CI: -1.80 to 5.30); equivalent to a change of 7.16% ±33.33 (95%CI: -8.44 to 22.7). Muscle force time course evolution is presented in the table just below the Fig. 1. Additional figures illustrating individual changes are presented in the supplemental data document (see e-Figs. 1 and 2, for normalized and raw muscle strength values). The two-way ANOVA for repeated measures revealed a significant interaction between muscle groups and time (*p* = 0.0017), indicating a difference between inspiratory and quadriceps muscles’ ability to develop a series of maximal contractions according to time. However, muscle group or time factor alone did not show any differences (respectively, *p* = 0.19 and *p* = 0.62). Still, the subject factor did (*p* < 0,0001), revealing inter-individual variability in muscle force time course evolution. While some patients showed a more pronounced decline in quadriceps force, others maintained a relatively stable performance. No statistically significant difference was found between muscle groups after comparing the deltas (inspiratory muscles delta M10-M1: 6.994% ±19.59; quadriceps muscle delta: 13.86% ±20.15; *p* = 0.17). Post-hoc analyses comparing the strength of muscle groups at different time points showed significant differences for the 8th, 9th, and 10th measurements (with *p* = 0.005; *p* = 0.0189 and *p* = 0.0276, respectively). An exploratory analysis was performed to assess a potential difference between patients who were able to take measurements before and after a mean of 9 days post-extubation. This analysis showed no statistically significant difference for the inspiratory or quadriceps muscles (see e-Fig. 3).

After a minute of passive recovery, quadriceps muscle’s force significantly increased from 86.31 ± 49.60 to 97.61 ± 61.57 N (mean difference: +14.25 N; (95%CI: 3.47 to 25.04); *p* = 0.0124), equivalent to a change of 13.8% ±20.15 (95%CI: 4.14 to 23.57). No statistically significant variation was found for Pimax: from 35.11 ± 17.40 to 38.00 ± 20.10 cmH_2_O (mean difference: +2.89 cmH_2_O; (95%CI: -0.92 to 6.70); *p* = 0.16), equivalent to a change of 7% ±19.59 (95%CI: -2.75 to 16.74).

## Discussion

Our study investigated the ability of inspiratory and quadriceps muscles of ICU-Aw patients presenting weakness at extubation to sustain repeated maximal contractions. Our main findings indicate: (1) a significant difference in the time course evolution of muscle strength between muscle groups: quadriceps muscle force significantly declined over repetitions whereas Pimax remained stable; (2) the ability of the quadriceps muscle to significantly recovered after a one-minute break, whereas no such change could be observed for the inspiratory muscles since strength remained stable over the ten repetitions.

Our main objective was to observe whether inspiratory and limb muscles might have different capacities to produce and maintain effort in the context of muscle impairment caused by ICU stay. Our results suggest a difference in the ability of the inspiratory and the quadriceps muscles to maintain strength during repeated effort. We observe this through the tendency for the force produced by the quadriceps to decrease and the maintenance of the Pi_max_. Previous studies have raised concerns that the ICU environment - characterized by prolonged sedation, controlled modes of ventilation, and the use of neuromuscular blockers - imposes a forced rest on muscles that are normally in constant use, particularly the inspiratory muscles. Unlike limb muscles, which are more accustomed to periods of inactivity, respiratory muscles are continuously active under normal conditions. This had led some authors to hypothesize that immobilization may disproportionately affect respiratory muscles and be associated with poor short-term outcomes [10].

It is important to note that the Pimax measurements we carried out take into account not only the activity of the diaphragm, which is the main muscle studied in the literature, but also the activity of the accessory inspiratory muscles. In a recent study to predict extubation failure, Dres and colleagues highlighted the variable activation of the accessory inspiratory muscles in an attempt to maintain inspiratory function in the presence of diaphragmatic dysfunction [23]. This could partly explain the stability of the muscle force observed: a decrease in diaphragm activation could be partially compensated by an increase in accessory muscle activation, thus maintaining function. Unfortunately, the Pimax performed in our study does not allow us to take into account the individual contribution of the inspiratory muscles.

We observed that the mean Pimax increased from < 30 cmH_2_O at the time of extubation to 32.6 cmH₂O at the time of the first muscle force measurement, and even to 38 cmH₂O after the one-minute recovery. This improvement is probably linked to the average delay of 9 days between extubation and measurement, the time needed for patients to reach IMS 4. It is possible that these 9 days on average enabled greater recovery of the respiratory muscles than the muscles of the limbs, due to the continued and cyclic activity of the inspiratory muscles after weaning from mechanical ventilation compared to limb muscles. Unfortunately, we are unable to quantify the progression of quadriceps strength during this period because reliable quadriceps dynamometry could not be performed earlier. This may explain why we observed a tendency for quadriceps strength to decline, while the inspiratory muscles maintained their ability to sustain repeated maximal efforts.

To date, no study has investigated the ability of weakened muscles to generate force or focused on their recovery after extubation in the ICU setting. The oxidative profile, together with the marked changes in muscle fiber damage, may suggest that inspiratory muscles may recover more rapidly than the limb muscles [24–27]. The difference in the ability of these muscles to recover can be explained by their different anatomy and physiology, as well as the nature of the injury itself. It is now well established that ICU-AW and respiratory weakness are distinct entities. Recent studies have shown that they do not always occur together, nor do they have the same long-term effects or prognosis [13, 14, 28].

Our results showed inter-individual variability in the time course evolution of maximal volitional strength, reinforcing the idea that rehabilitation might consider patients’ individual characteristics. While no curative therapy exists for ICU-AW, early sepsis treatment, minimum sedation, glycemic control, and physiotherapy may decrease its incidence [1, 9, 29]. We still have no precise idea of which rehabilitation programs to propose for ICU-AW patients, regarding which muscle group to prioritize, which is the most likely to recover, what type of exercise to perform, when, and with which patient; while desperately needing strategies to impact short and long-term outcomes. But these findings have implications for ICU rehabilitation strategies: the differential response of respiratory and limb muscles suggests that rehabilitation protocols should be tailored to address the specific needs of each muscle group. Since quadriceps muscles show a greater decline in force over repeated efforts, interventions may need to focus on strengthening and endurance exercises for peripheral muscles. Meanwhile, respiratory muscle training might prioritize maintaining endurance without overloading patients in the early stages of recovery.

There are several limitations to this study that need to be taken into consideration. Firstly, this was a single-center study in a medical ICU with a very limited number of patients. No a priori sample size calculation was made, so we cannot rule out the possibility that this study was underpowered and therefore failed to highlight changes in muscle force evolution over the ten repetitions. However, given the lack of data in the literature, this study provides an initial insight into the behaviour of these muscles. As 90% of our patients were men, it is difficult to generalise the results to a general population in intensive care, but further analysis of such a small number of patients would be pointless. 

Secondly, we tried to standardize muscle evaluation as much as possible, including resistance applied, position, and duty cycle. However, we cannot exclude the possibility that the tools used were largely dependent on the patient’s will. The potential variation in muscle strength identified might, in fact, be linked to a sub-maximal contraction due to pain, fatigue, fluctuating participation. Nevertheless, these factors can influence maximal strength during rehabilitation processes and should be considered. While the decline in quadriceps muscle strength related to our protocol was anticipated, it is encouraging to observe that the inspiratory muscles maintain their strength.

The third limit is the delay between our muscle strength measurements and mechanical ventilation weaning. On average, it took 9 days after extubation for patients to be able to stand with assistance (ICU mobility scale = 4) and sit in a chair, and therefore for measurements to be undertaken. However, considering an ICU Mobility Scale of 4 and a Pi_max_ of 32 cmH_2_O, the patients were still weak at the time of measurement. We attempted to investigate the effect of this delay of 9 ± 4 days on our results and to compare patients according to the time between extubation and measurements. This exploratory analysis did not show any statistical differences, but further research is needed to identify trends in muscle recovery of dysfunction after extubation.

Finally, since our fatigue protocol has never been applied in the ICU, we arbitrarily chose to have the patients perform 10 repetitions at 100% of their maximal strength, and we do not know if it would have been better to choose another method. It is difficult to say whether a longer, more intense program would have produced more significant differences. From a clinical point of view, performing ten repetitions was already very difficult for all the participants.

## Conclusions

Our study highlighted the difference in the ability to sustain repeated maximal voluntary contractions between the respiratory and limb muscles in ICU-AW patients: the inspiratory muscles appear to be more resistant than the quadriceps, consistent with the well-established idea that ICU-AW and respiratory weakness are distinct conditions. These observations remain to be confirmed, and further research with large-scale studies is needed to investigate the evolution of muscle function over time to guide the development of tailored rehabilitation strategies to prevent muscle deterioration and improve functional recovery.

## Electronic supplementary material

Below is the link to the electronic supplementary material.

Supplementary Material 1

## Data Availability

The datasets generated and/or analyzed during the current study are not publicly available but are available from the corresponding author on reasonable request.

## Declarations

### Authors’ information

The study was performed in Le Havre Hospital, F-76600, Le Havre, France. This paper was presented as poster at the ERS Congress that was held in Vienna on the 8th of September, 2024.
