# Supplementary material for: Respiratory and limb muscles’ ability to repeatedly generate maximal isometric strength in patients with intensive care unit-acquired weakness: an observational study
Source: BMC Anesthesiol. 2025 Mar 20;25:134. doi: 10.1186/s12871-025-03008-y (PMC11924868; doi:10.1186/s12871-025-03008-y)
Supplement: Supplementary file 1 — Supplementary Material 1 [file 12871_2025_3008_MOESM1_ESM.pdf]

Respiratory and limb muscles' ability to repeatedly generate maximal force in patients with intensive care unit-acquired weakness: an observational study

Margaux MACHEFERT, Guillaume PRIEUR, Solène AUBRY, Yann COMBRET, Clément MEDRINAL

Supplemental material

Supplemental Figures

e-Figure1. Muscle force time course evolution over the ten repetitions, with individual data expressed for both muscle groups in % of first contraction.

e-Figure2. Muscle force time course evolution over the ten repetitions, with individual data expressed in unit of force, centimeter of water (cmH_2O) for Pimax and Newtons (N) for quadriceps muscle maximal strength.

e-Figure3. Muscle force time course evolution over the ten repetitions according to time delay between extubation and measurements, with data expressed for both muscle groups in % of first contraction. Panel A: for quadriceps muscle; Panel B: for inspiratory muscles.

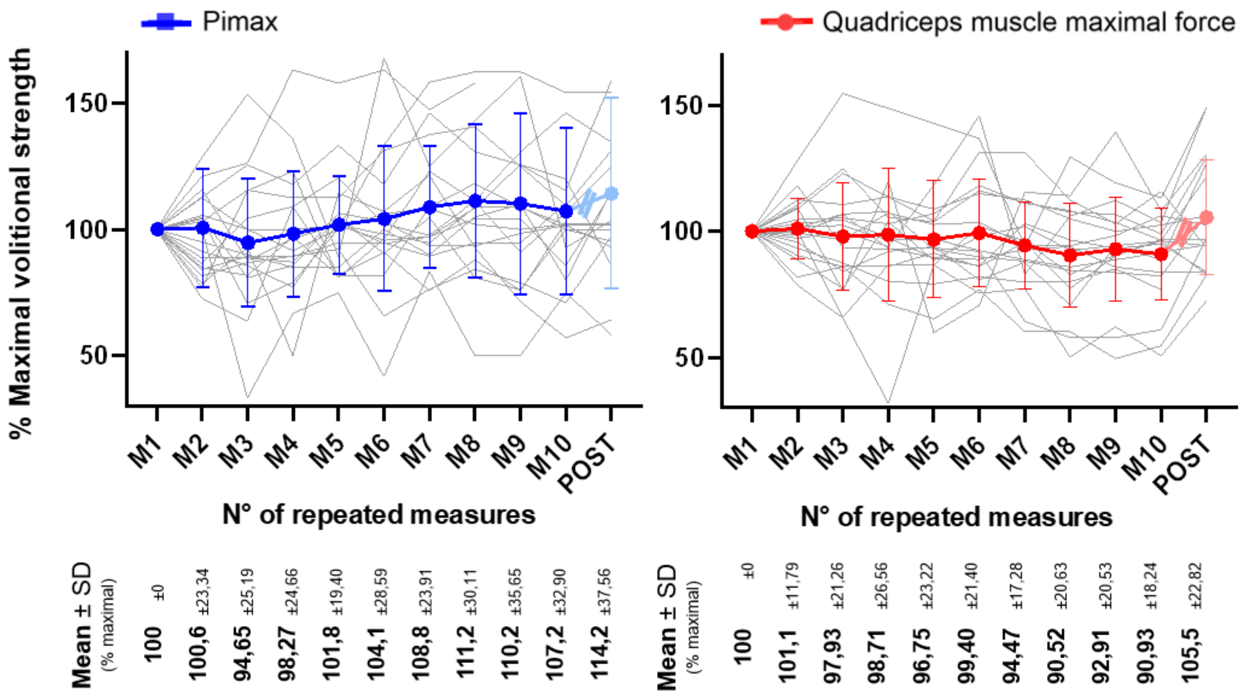

e-Figure1. Muscle force time course evolution over the ten repetitions, with individual data expressed for both muscle groups in % of first contraction. SD: standard deviation.

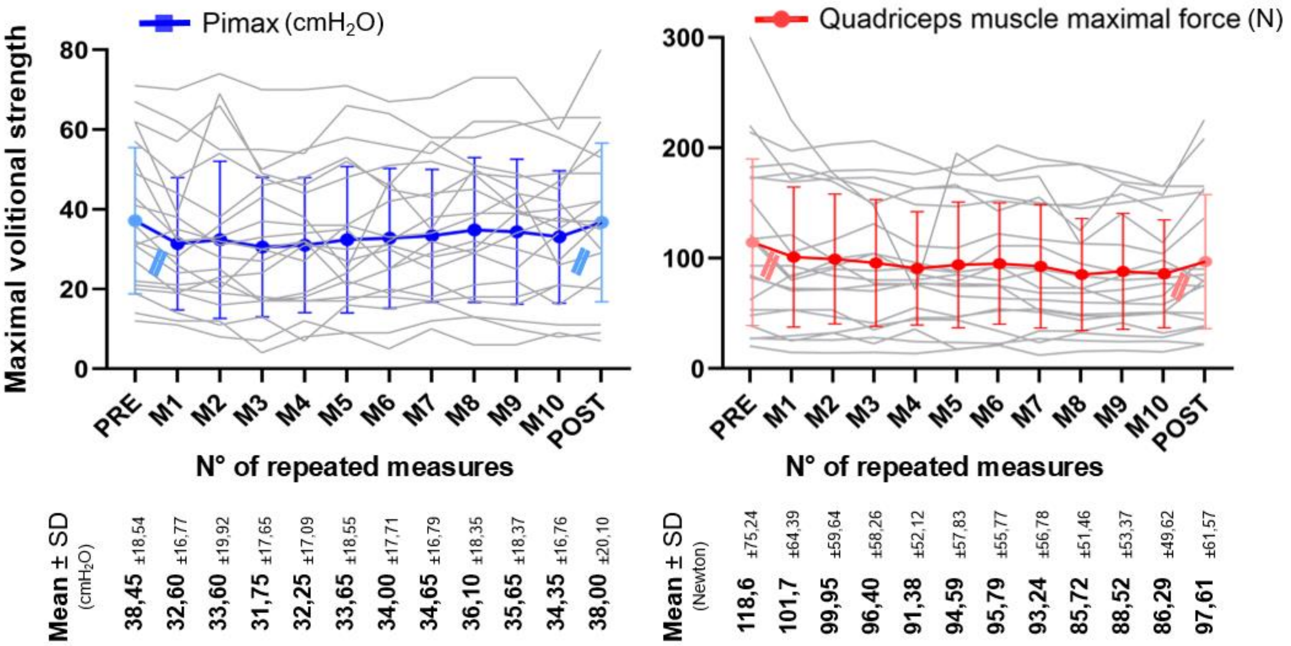

e-Figure2. Muscle force time course evolution over the ten repetitions, with individual data expressed in unit of force, centimeter of water (cmH_2O) for Pimax and Newtons (N) for quadriceps muscle maximal strength. SD: standard deviation.

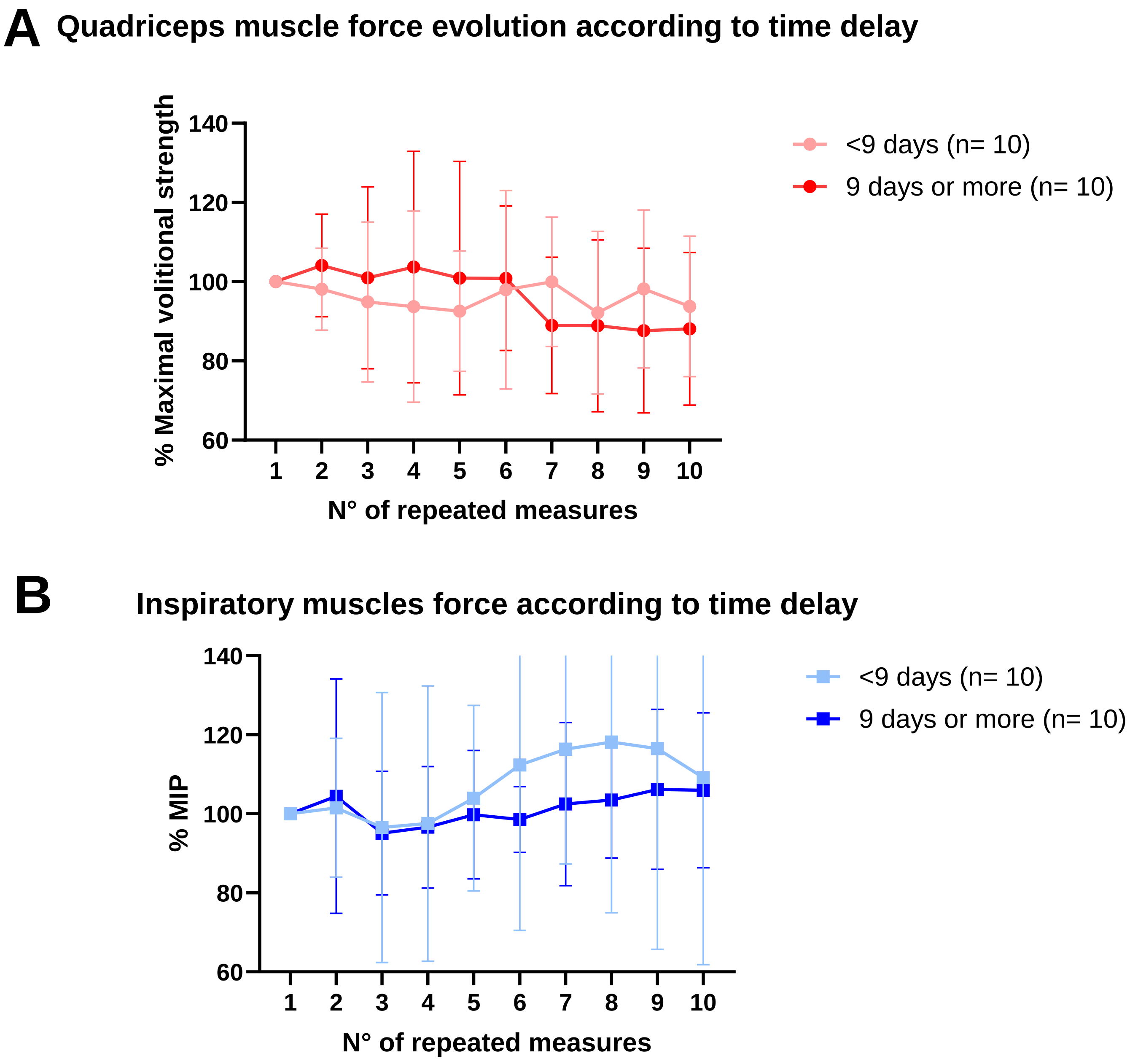

e-Figure3. Muscle force time course evolution over the ten repetitions according to time delay between extubation and measurements, with data expressed for both muscle groups in % of first contraction. Panel A: for quadriceps muscle; Panel B: for inspiratory muscles. * Indicates statistically significant between-group differences.
